# Impact of peer review on discussion of study limitations and strength of claims in randomized trial reports: a before and after study

**DOI:** 10.1186/s41073-019-0078-2

**Published:** 2019-09-16

**Authors:** Kerem Keserlioglu, Halil Kilicoglu, Gerben ter Riet

**Affiliations:** 1 Department of General Practice, Amsterdam Public Health Research Institute, Amsterdam UMC, University of Amsterdam, Meibergdreef 9, 1105AZ Amsterdam, The Netherlands; 2 Lister Hill National Center for Biomedical Communications, U.S. National Library of Medicine, Bethesda, MD USA; 3 Department of Cardiology, Amsterdam UMC, University of Amsterdam, Meibergdreef 9, 1105AZ Amsterdam, The Netherlands; 4 ACHIEVE Centre for Applied Research, Amsterdam University of Applied Sciences, Tafelbergweg 51, 1105 BD Amsterdam, The Netherlands

**Keywords:** Peer review, Study limitations, Before-after study, Linguistic spin, Hedging, Transparency, Scientific reporting, Randomized trial

## Abstract

**Background:**

In their research reports, scientists are expected to discuss limitations that their studies have. Previous research showed that often, such discussion is absent. Also, many journals emphasize the importance of avoiding overstatement of claims. We wanted to see to what extent editorial handling and peer review affects self-acknowledgment of limitations and hedging of claims.

**Methods:**

Using software that automatically detects limitation-acknowledging sentences and calculates the level of hedging in sentences, we compared the submitted manuscripts and their ultimate publications of all randomized trials published in 2015 in 27 BioMed Central (BMC) journals and BMJ Open. We used mixed linear and logistic regression models, accounting for clustering of manuscript-publication pairs within journals, to quantify before-after changes in the mean numbers of limitation-acknowledging sentences, in the probability that a manuscript with zero self-acknowledged limitations ended up as a publication with at least one and in hedging scores.

**Results:**

Four hundred forty-six manuscript-publication pairs were analyzed. The median number of manuscripts per journal was 10.5 (interquartile range 6–18). The average number of distinct limitation sentences increased by 1.39 (95% CI 1.09–1.76), from 2.48 in manuscripts to 3.87 in publications. Two hundred two manuscripts (45.3%) did not mention any limitations. Sixty-three (31%, 95% CI 25–38) of these mentioned at least one after peer review. Changes in mean hedging scores were negligible.

**Conclusions:**

Our findings support the idea that editorial handling and peer review lead to more self-acknowledgment of study limitations, but not to changes in linguistic nuance.

## Background

One of the main functions of the editorial process (peer review and editorial handling) as employed by almost all serious scientific journals is to ensure that the research articles published are accurate, transparent, and complete reports of the research conducted. *Spin* is a term used to describe reporting practices that distort the interpretation of a study’s results [1]*.* Not mentioning (all important) study limitations is one way in which readers can be misguided into believing that, for example, the beneficial effect of an experimental treatment is greater than the trial’s result warrant.

In a survey among scientists, insufficient reporting of study limitations ranked high in a list of detrimental research practices [2]. In a masked before-after study at the editorial offices of Annals of Internal Medicine, Goodman et al. found that the reporting of study limitations was fairly poor in manuscripts but improved after peer review and editing [3]. Ter Riet et al. demonstrated that more than a quarter of biomedical research articles do not mention any limitations [4]. And finally, Horton, in a survey among all authors of ten Lancet papers, found that “Important weaknesses were often admitted on direct questioning, but were not included in the published article” [5]. Other forms of *spin* are inappropriate extrapolation of results and inferring causal relationships when the study’s design does not allow for it [1].

Peer reviewers should spot and suggest changes to overstatements and claims that are too strong and point out non-trivial study weaknesses that are not mentioned. The peer review process may therefore been seen as “a negotiation between authors and journal about the scope of the knowledge claims that will ultimately appear in print” [6]. Specific words that can be used to add nuance to statements and forestall potential overstatement are so-called “hedges”; these are words like “might,” “could,” “suggest,” “appear,” etc. [7] Authors of an article are arguably in the best position to point out their study’s weaknesses, but they may feel that naming too many or discussing them too extensively could hurt their chances of publication. In this contribution, we hypothesized that, compared to the subsequent publications, the discussion sections of the submitted manuscripts contain fewer acknowledgments of limitations and are less strongly hedged.

## Methods

In this study, we considered the discussion sections of randomized clinical trial (RCT) reports published in 27 BioMed Central (BMC) journals and BMJ Open. Using two software tools, we determined the number of sentences dedicated to the acknowledgment of specific study limitations and the use of linguistic hedges, before (manuscripts) and after peer review (publications). The limitation detection tool relies on the structure of the discussion sections and linguistic clues to identify limitation sentences [8]. In a formal evaluation, its accuracy was found to be 91.5% (95% CI 90.1–92.9). The hedging detection tool uses a lexicon containing 190 weighted hedges. The system computes an overall hedging score based on the number and strength of hedges in a text. Hedge weights range from 1 (low hedging strength, e.g., “largely”) to 5 (high hedging strength, e.g., “may”). The overall hedging score is then divided by the word count of the discussion section (normalization). We also calculated “unweighted” scores, in which all hedges are weighted equally as 1. The software tool yielded 93% accuracy in identifying hedged sentences in a formal evaluation [9]. The manuscripts were downloaded from the journals’ websites followed by manual pre-processing to restore sentence and paragraph structure. Our software automatically extracted the discussion sections in the publications from PubMed Central.

We also carried out a qualitative analysis of the two publications with the largest increase and decrease of hedging score, respectively. For these two papers, KK compared the before and after discussion sections to see what the actual changes were. The reviewer reports, consisting of the reviewer’s comments and the authors responses, were analyzed.

We performed mixed linear regression analysis, for each manuscript-publication pair, of the mean changes in the number of limitation sentences and normalized hedging scores, with the journal as a random intercept. We repeated these analyses adjusting for the journal’s impact factor (continuous), editorial team size (continuous), and composition of authors in terms of English proficiency (three dummy variables representing four categories). English proficiency was derived from the classification of majority native English-speaking countries by the United Kingdom (UK) government for British citizenship application [10]. English proficiency was categorized as follows: (i) All authors are residents of an English native country; (ii) the first author is an English native, but at least one co-author is not; (iii) the first author is not an English native, but at least one co-author is; and (iv) none of the authors are English natives. We performed a sensitivity analysis, in which we excluded the manuscript-publication pairs of BMJ Open (*n* = 69) and BMC Medicine (*n* = 14) due to their exceptional number of editorial team members (84 and 182, respectively). Finally, using scatterplots and fractional polynomial functions, we visually explored if the effect on the changes in the number of limitation-acknowledging sentences was affected by the number of limitation-acknowledging sentences in the manuscript controlled for regression to the mean using a median split as suggested by Goodman et al. [3]. We present the results of the crude and adjusted analyses in Table 2 and those of the sensitivity analyses in Appendix 1.

We used mixed-effects logistic regression analysis to assess the impact of the abovementioned factors on the likelihood of mentioning at least one limitation in the publication among those that had none in the manuscript. Sensitivity analyses consisted of restricting the data set to the journals with fewer than 20 editorial team members, at least 10 manuscript-publication pairs, and both of those restrictions simultaneously, respectively.

## Results

**Fig. 1:**
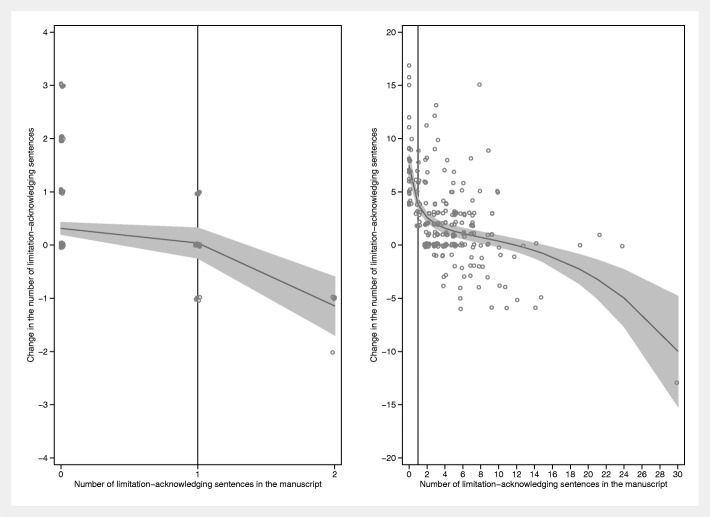
Changes in the number of limitation-acknowledging sentences between manuscripts and publications as a function of the number such sentences in the manuscript. Left panel: manuscript-publication pairs below the median split. Right panel: manuscript-publication pairs above the median split. The median split was calculated as the average of the number of limitation-acknowledging sentences in the manuscript (*L*_*m*_) and in the publication (*L*_*p*_): (*L*_*m*_ + *L*_*p*_)/2. These averages were ranked and the median (value = 2; interquartile range 0–5) determined. The lines are fitted using fractional polynomials with 95% confidence intervals (Stata 13.1, *twoway fpfitci* command). Note that, in both panels, the changes tend to increase with decreasing numbers of limitation-acknowledging sentences in the manuscript. In particular, the effect of peer review and editorial handling is large in those manuscripts above the median split (right panel) with zero limitation-acknowledging sentences in the manuscript. The vertical line in the right panel is the line *x* = 1. Cluttering of data points was prevented by jittering them. Therefore, data points for *x* = 0 are not placed exactly above the tick mark for *x* = 0 but somewhat scattered to the left and right. The same holds for all data points and for the vertical placement of the points

Four hundred forty-six research articles were selected. Table 1 shows a few key journal characteristics. The median number of manuscripts per journal was 10.5 (interquartile range (IQR) 6.5–18.5; range 2–69). Table 2 shows the results. The average number of distinct limitation sentences increased by 1.39, from 2.48 (manuscripts) to 3.87 (publications). Two hundred two manuscripts (45.3%) did not mention any limitations. Sixty-three (31%, 95% CI 25–38) of these mentioned at least one after peer review. Of the 244 manuscripts that mentioned at least one limitation, eight (3%, 95% CI 2–6) mentioned none in the publication. Across the (sensitivity) analyses performed, the probability of mentioning at least one limitation in the publication among those that had none in the manuscript was not consistently associated with any of the three covariables assessed, although higher impact factors tended to be weakly associated with lower probabilities and size of the editorial team weakly with higher probabilities (data not shown). The visual assessment of how the number of changes in the limitation-acknowledging sentences depended on the number of such sentences in the manuscript showed an inverse relation, that is, larger changes were seen in manuscripts with low numbers of limitation-acknowledging sentences (Fig. 1).

**Table 1:** Journal characteristics

	BMC (*N* = 377)	BMJ Open (*N* = 69)
Journal characteristic		
Peer review type	Open	Open
Acceptance rate (%, range)	45–55	55
Impact factor*	2.10 (1.66, 2.29)	2.56
Size editorial team*	8 (6, 16)	84
Days until publication*	196 (141, 270)	192 (149, 225)

*median (interquartile range)

**Table 2:** The results of the crude and adjusted analyses

	Manuscript	Publication	Crude difference or proportion^‡^ (95% CI)	Adjusted difference^†^ (95% CI)
Number of limitation-acknowledging sentences (mean, SD)	2.48 (3.62)	3.87 (4.34)	1.39 (1.09–1.76)	0.62 (− 0.23–1.48)
Number of papers with zero limitation-acknowledging sentences (*n*/total)	202/446	147/446		
Number of manuscripts with zero limitation-acknowledging sentences whose publication had at least one	63/202		31.2 (25.2–37.9)	
Number of manuscripts with at least one limitation-acknowledging sentence whose publication had none		8/244	3.28 (1.67–6.34)	
Unweighted hedges (%)	2.06 (0.76)	2.13 (0.74)	0.07 (0.04–0.10)	0.04 (− 0.05–0.14)
Unweighted hedges, limitation-acknowledging sentences excluded (%)	2.01 (0.77)	2.05 (0.76)	0.04 (0.01–0.08)	0.06 (− 0.03–0.16)
Weighted hedges (%)	7.07 (2.91)	7.30 (2.82)	0.23 (0.10–0.36)	0.09 (− 0.28–0.47)
Weighted hedges, limitation-acknowledging sentences excluded (%)	6.92 (2.95)	7.05 (2.89)	0.13 (0.01–0.26)	0.05 (− 0.32–0.43)

*N* = 440 because we were unable to find the impact factor of BMC Dermatology (contributing six manuscript-publication pairs); hedges were counted (and weighted), divided by the total number of words in the discussion section and multiplied by 100

*SD* standard deviation

^‡^Crude difference estimated using a mixed regression model without covariables (*N* = 446)

^†^Adjusted for journal impact factor (continuous), editorial team size (continuous), and composition of authors in terms of English proficiency (three dummy variables)

The hedging-related differences were all very close to zero. A post hoc analysis inspired by the hypothesis that limitation-acknowledging sentences themselves might affect the average hedging scores confirmed the main analysis.

The largest increase in hedging score was + 1.67 (from 3.33 to 5.00). The weighted hedging scores were 50 across the 15 detected sentences in the manuscript and 145 across the 29 detected sentences in the published paper, respectively. The largest decrease in hedging score was − 2.55 (from 6.85 to 4.30). The weighted hedging score was 192 across 28 sentences in the manuscript and 142 across 33 sentences in the published paper (see Appendix 3 for the textual changes).

## Discussion

In a sample of 446 randomized trial reports published in 28 open access journals, we found a 56% increase in the number of sentences dedicated to study limitations after peer review, although one may argue that in absolute terms, the gain was modest (1.39 additional sentences). Our automated approach showed that 33% of research reports do not contain limitation sentences after peer review. This is comparable with the finding of 27% by Ter Riet et al., which they determined with a manual approach. Goodman et al. found that mentioning study limitations is one of the poorest scoring items before and one of the most improved factors after peer review [3]. Like Goodman et al., we found evidence that peer review and editorial handling had greater impact on manuscripts with zero and very low numbers of limitation-acknowledging sentences. In Appendix 2, we highlight the attention to mentioning study limitations in seven major reporting guidelines.

Our findings do not support the hypothesis that the editorial process increases the qualification of claims by using a more nuanced language. The small-scale qualitative analysis of two manuscript-publication pairs indicated that authors are asked to both tone down statements, that is, hedge more strongly, and make statements less speculative, that is hedge less. These phenomena may offset each other resulting in minimal changes in the overall use of hedges (see Appendix 3 for the actual text changes). While the hedging terms and their strength scores were selected based on a careful analysis of the linguistic literature on this topic, it is possible that authors use terms indicating different degrees of certainty (e.g., *could* vs. *may*) somewhat interchangeably. This may explain our finding that the net change in hedging scores was very small.

To better understand the influence of peer review on changes made to manuscripts before publication, it may be interesting to conduct more extensive qualitative analyses of the peer review reports and correspondence available in the files of editorial boards or publishers. Another interesting research avenue may be the comparison of rejected manuscripts to accepted ones, to assess if acknowledgment of limitations and degree of hedging affects acceptance rates. It may be useful to restrict such analyses to sentences in which particular claims on, for example, generalizability are made.

Arguably, our software tools might be utilized by editorial boards (or submitting authors) to flag up particular paragraphs that might deserve more (editorial) attention. The limitation sentence recognizing software could for example be used to alert editors to manuscripts with zero self-acknowledged limitations to see if such omission can be justified. If reference values existed that represented the range of hedging scores across a large body of papers, the hedge-detection software could help inform reviewers (or even authors) that the manuscript has an unusual (weighted) hedging score and let them revisit some the formulations in the paper. We think that currently, no direct conclusions should be drawn from the numbers alone. Human interpretation will remain critical for some time to come, but a signposting role of the software seems currently feasible.

A limitation of our study is that we only included reports or randomized trials that made it to publications. Acknowledgment of limitations among all submissions, including also observational studies, may be different than what we report here. Another limitation is that we only included open peer review journals of more than average editorial team quality. Blind peer review may lead to different results as may the case for journals with lower quality editorial team. Note also that the weight assigned to the hedges is somewhat subjective. However, our results were stable across weighted and unweighted hedges. Finally, one may argue that there is a discrepancy between our interest in overstated claims and what we actually measured, namely, hedging scores in *all* sentences in the discussion sections. A stricter operationalization of our objective would have required that we detect “claim sentences” first and then measure hedging levels in those sentences only. On the other hand, our approach to focus on discussion sections only is better than analyzing complete papers, because claims are usually made in the discussion sections. A strength of our study is the automated assessment of limitation sentences and hedges, limiting the likelihood of analytical or observational bias. Such automated assessment could also assist journal editors as well as peer reviewers in their review tasks. Our results suggest that reviewers and/or editors demand discussion of study limitations that authors were unaware of or unwilling to discuss. Since good science implies the full disclosure of issues that may (partially) invalidate the findings of a study, this increase in the number of limitation sentences is a positive effect of the peer and editorial review process.

## Conclusion

Our findings support the idea that editorial handling and peer review, on average, cause a modest increase in the number of self-acknowledged study limitations and that these effects are larger in a manuscript reporting zero or very few limitations. This finding is important in the debates about the value of peer review and detrimental research practices. Software tools such as the ones used in this study may be employed by authors, reviewers, and editors to flag potentially problematic manuscripts or sections thereof. More research is needed to assess more precisely the effects, if any, of peer review and editorial handling on linguistic nuance of claims.

## Data Availability

The datasets used and/or analyzed during the current study as well as the software tools used for the detection of limitation-acknowledging sentences and hedges are available from the corresponding author on reasonable request.

## Appendix 1

**Table 3:** Sensitivity analyses

	Manuscript	Publication	Crude difference^‡^ (95%CI)	Adjusted difference^†^ (95%CI)
Number of limitation-acknowledging sentences (mean, SD)	2.61 (3.81)	3.96 (4.51)	NA	1.74 (0.21–3.28)
Unweighted hedges (%)	2.06 (0.76)	2.13 (0.74)	0.06 (0.03–0.10)	− 0.01 (− 0.18–0.15)
Unweighted hedges, limitation-acknowledging sentences excluded (%)	2.01 (0.77)	2.05 (0.76)	0.04 (0.01–0.08)	0.06 (− 0.03–0.16)
Weighted hedges (%)	7.07 (2.91)	7.30 (2.82)	0.20 (0.07–0.34)	0.03 (− 0.62–0.26)
Weighted hedges, limitation-acknowledging sentences excluded (%)	6.92 (2.95)	7.05 (2.89)	0.13 (0.01–0.26)	0.08 (−0.58–0.74)

Results obtained via the same calculations as in Table 2, but excluding BMJ Open and BMC Medicine whose editorial team sizes were extremely large compared to the other 26 journals, namely, 84 and 182, respectively. After omitting these two journals, the median team size was 8 (*IQR* interquartile range; IQR 6–14). *N* = 357 because we were unable to find the impact factor of BMC Dermatology (contributing six manuscript-publication pairs); hedges were counted (and weighted), divided by the total number of words in the discussion section and multiplied by 100. *NA* not available, since that model did not converge and no coefficients were calculated

*SD* standard deviation

^‡^Crude difference estimated using a mixed regression model without covariables (*N* = 363);

^†^Adjusted for journal impact factor (continuous), editorial team size (continuous) and composition of authors in terms of English proficiency (three dummy variables)

## Appendix 2

**Table 4:** Examples of what seven major reporting guidelines state about the need to mention limitations

Reporting guideline	Suggestions pertaining to self-acknowledgment of limitations
CONSORT	Trial limitations, addressing sources of potential bias, imprecision, and, if relevant, multiplicity of analyses; generalizability (external validity, applicability) of the trial findings; interpretation consistent with results, balancing benefits and harms, and considering other relevant evidence
CONSORT 2010 statement: extension to randomized pilot and feasibility trials	Pilot trial limitations, addressing sources of potential bias and remaining uncertainty about feasibility; generalizability (applicability) of pilot trial methods and findings to future definitive trial and other studies; interpretation consistent with pilot trial objectives and findings, balancing potential benefits and harms, and considering other relevant evidence. Implications for progression from pilot to future definitive trial, including any proposed amendments
Reporting of stepped wedge cluster randomized trials	Trial limitations, addressing sources of potential bias, imprecision, and, if relevant, multiplicity of analyses; generalizability (external validity, applicability) of the trial findings. Generalizability to clusters or individual participants, or both (as relevant); interpretation consistent with results, balancing benefits and harms, and considering other relevant evidence.
CONSORT extension for reporting N-of-1 trials (CENT) 2015 Statement	Trial limitations, addressing sources of potential bias, imprecision, and, if relevant, multiplicity of analyses; generalizability (external validity, applicability) of the trial findings; interpretation consistent with results, balancing benefits and harms, and considering other relevant evidence
PRISMA Preferred Reporting Items for Systematic Reviews and Meta-Analyses	Discuss limitations at study and outcome level (e.g., risk of bias), and at review-level (e.g., incomplete retrieval of identified research, reporting bias); provide a general interpretation of the results in the context of other evidence, and implications for future research
The Strengthening the Reporting of Observational Studies in Epidemiology (STROBE) Statement	Discuss limitations of the study, taking into account sources of potential bias or imprecision; discuss both direction and magnitude of any potential bias; interpretation gives a *cautious overall interpretation* of results considering objectives, *limitations*, multiplicity of analyses, results from similar studies, and other relevant evidence. Discuss the generalizability (external validity) of the study results
TRIPOD: Transparent reporting of a multivariable prediction model for individual prognosis or diagnosis	Discuss any limitations of the study (such as non-representative sample, few events per predictor, missing data); interpretation: for validation, discuss the results with reference to performance in the development data and any other validation data. Give an overall interpretation of the results, considering objectives, *limitations*, results from similar studies, and other relevant evidence

Text was abstracted via http://www.equator-network.org/reporting-guidelines/consort-cent/, which was accessed on 12 June 2019

## Appendix 3

### Qualitative analysis

The two articles with the largest increase and decrease in their discussion sections’ hedging scores after the editorial process were evaluated. We analyzed the before and after discussion sections as well as the correspondence between reviewers and authors.

The largest increase in a discussion section hedging score was + 1.67 (from 3.33 to 5.00).

There was one clear example of the adding of a hedge.

Manuscript: *The present study suggests that there is no evidence of an effect of the completion of a standard informed consent procedure on…*

Publication: *The present study suggests that there is* *no strong* *evidence of an effect of the completion of a standard informed consent procedure on…*No other sentences were adjusted. However, three paragraphs were added, mainly consisting of study limitations and nuancing of findings:

Publication: *If effects of the type we hypothesised do exist, and we suggest that despite the overall finding, this study can provide some tentative evidence that they do, we may anticipate that they will vary in their magnitude…*Reviewer’s comment: “The authors mention in their response that they don’t have access to timing. While I appreciate that, there should be better discussion on this broader point in the manuscript. For example, how would the authors have done things differently? One possibility is to ask a question of the participants who received the intervention that, perhaps indirectly, evaluates whether or not they read the information sheet. In the discussion, the authors have an opportunity to be a little creative in what they suggest.”

Author’s response: “This is very helpful and is now discussed towards the end of the discussion section.”

Publication: *Another limitation of the study is that we are unable to determine if the participants assigned to the intervention group actually read the information… Although we could have adopted strategies such as … The absence of any exposure enhancement measures in the present study, also implies some degree of experimental manipulation failure, in that not all randomised participants may have been fully exposed to the possible effects we were seeking measure. This should be borne in mind when interpreting the results of the present study*.The largest decrease in a discussion section hedging score was − 2.55 (from 6.85 to 4.30).

Manuscript: *Our results contrast with previous animal data by indicating that RIC appears to be an effective adjunct to pPCI in STEMI patients regardless of most cardiovascular risk factors…*Reviewer’s comment: “In my opinion there is some over-interpretation in the Discussion. The opening statement that RIC appears to be an effective adjunct to pPCI in STEMI patients is based on a confidence interval with a lower limit of 0. This is of borderline statistical significance.”

Author’s response: “We have revised as recommended and down-graded the opening statement. Additionally, we have specified that the statistical power was limited, and our study should be considered exploratory.”

Publication: *Our analysis did not demonstrate significant modification on the efficacy of RIC by cardiovascular risk factors and their medications in patients with STEMI undergoing pPCI. Because the statistical power was limited, our study should be considered exploratory.*Reviewer’s comment: “Within each of the subgroups in the discussion, as tests for interaction have not been performed the interpretation is somewhat subjective as to whether there is a difference in RIC effect between the subgroups. In places I feel the interpretation is too strong, and this part of the discussion is too long.”

Manuscript: *In our clinical randomised study, we did not find an attenuated effect of RIC in patients with diabetes mellitus or in patients with high plasma glucose or HbA1c levels. Rather, the point estimates tended to support the opposite effect. Antidiabetic drugs may modulate the response to RIC, but because of the limited number of diabetic patients in our study, we were unable to stratify our analysis according to type of antidiabetic treatment.*

Publication: *The number of patients with diabetes mellitus was limited and our analysis does not allow a conclusion about the modification of the efficacy of RIC in patients with diabetes mellitus.*

Manuscript: *Our analysis demonstrated that the effect of RIC was preserved among statin users. Our data even may indicate that statin use increased the efficacy of RIC, as suggested by the markedly higher point estimate among statin users, although the confidence intervals were wide. Furthermore, we found that efficacy of RIC was independent of lipid levels at hospital admission.*

Publication: *Little is known about the effect modification of statin use on RIC. Thus, we are the first to indicate a potential increased effect of RIC in statin users. Whether RIC has a more pronounced effect in statin users deserves further investigation.*

Manuscript: *It would be instructive to investigate whether RIC and acute beta blocker treatment have additive cardioprotective effects. ACE inhibitors and ARBs have been shown to protect against reperfusion injury in animal models. However, angiotensin II also may be involved in the signaling cascade of ischaemic preconditioning. In a rabbit model, inhibition of the angiotensin II receptor (subtype AT1) with losartan eradicated the effect of local ischaemic conditioning. No studies have investigated the interaction of ACE inhibitor and ARB treatment with RIC, which may act through pathways other than local ischaemic preconditioning. Neither ACE inhibitors nor ARBs seemed to diminish the effect of RIC in our analysis, but additional animal and clinical studies are needed to clarify any potential modifying effect of ACE inhibitor and ARB treatment on RIC.*

Publication: This paragraph was removed entirely.

## Abbreviations

BMC: BioMed Central
CI: Confidence interval
IQR: Interquartile range
RCT: Randomized clinical trial
SD: Standard deviation
UK: United Kingdom
